# Experiences of Cancer-Related Cognitive Impairment Across Cancer Types: Qualitative Systematic Review

**DOI:** 10.2196/71996

**Published:** 2025-10-31

**Authors:** Maryam Ibrar, Harleen Kaur Rai, Ashleigh Main, Haruno McCartney, Mario A Parra, Roma Maguire

**Affiliations:** 1 University of Strathclyde Glasgow United Kingdom; 2 Trimbos Institute Utrecht The Netherlands

**Keywords:** cancer survivorship, cancer-related cognitive impairment, chemobrain, chemotherapy late effects, cognitive dysfunction, CRCI, return to work

## Abstract

**Background:**

Cancer-related cognitive impairment (CRCI) is frequently reported during cancer treatment, with 35% of patients experiencing cognitive issues even after treatment completion. Commonly reported impairments include difficulties with memory, attention, executive function, and processing speed, which often reduce daily functioning and quality of life (QoL). Despite its prevalence, CRCI remains underresearched across various cancer types, limiting understanding of the patient experience.

**Objective:**

This study aims to synthesize the qualitative evidence regarding the lived experience of CRCI across cancer types. It seeks to do so by exploring how commonly CRCI is subjectively experienced among cancer populations. It also aims to explore the cognitive domains perceived as most affected and the impact on QoL and functional ability.

**Methods:**

We conducted a qualitative systematic review using the PRISMA (Preferred Reporting Items for Systematic Reviews and Meta-Analyses) methodology. A comprehensive search across PubMed, APA PsycInfo, CINAHL, and Scopus for studies published from 2013 through July 2025 was performed. Articles addressing the experience of CRCI using qualitative or mixed methods were included. Two independent reviewers screened titles, abstracts, and full texts, with a third reviewer resolving conflicts during the inclusion process. Two reviewers piloted the data extraction process by discussing and selecting 10% of the studies. The Critical Appraisal Skills Programme checklist was used for data extraction and quality assessment. Data were analyzed using thematic analysis.

**Results:**

The database search identified 11,269 papers; 51 were included for analysis. Breast cancer was the most represented cancer type (n=32). The analysis revealed 4 themes. “Cognitive challenges” explores the impacted cognitive domains such as memory, concentration, executive functions, language, and processing speed; “navigating employment” discusses challenges associated with return to work, support, and disclosure; “emotional, behavioral, and psychological impacts” explores emotional and psychological responses; and “support systems” emphasizes the role of social and health care support. CRCI negatively affects QoL and functional ability, and there is lack of understanding and resources in place to manage its effects.

**Conclusions:**

This qualitative systematic review highlights the significant disruption of CRCI in daily life, stressing the need for increased awareness, standardized screening, and further research into digital interventions. Improved management of CRCI can support survivors of cancer in reintegrating into their daily lives and employment.

**International Registered Report Identifier (IRRID):**

RR2-10.2196/56888

## Introduction

### Overview

The worldwide incidence of cancer is rapidly increasing, with an estimated 19.3 million new cases and 10 million fatalities yearly [1]. Advancements in cancer therapies have significantly improved survival, but therapies such as chemotherapy, radiotherapy, and targeted cancer drugs remain associated with a range of adverse effects, including cognitive changes [2,3]. Approximately 75% of patients experience measurable cognitive impairment during treatment, and 35% continue to experience cognitive issues for months or even years after completing treatment [4].

Difficulties with memory, attention, executive function, and information processing speed are frequently reported by people with cancer [5]. These symptoms, linked to cancer treatment, are known as cancer-related cognitive impairment (CRCI), chemobrain, or chemotherapy-induced cognitive impairment [6] and are referred to in this study as CRCI. Given the lack of consensus on a standard definition in the literature, we have adopted a commonly used working definition: “a decline in cognitive function associated with cancer or its treatment, as reported by patients or measured objectively” [7]. This definition encompasses the subjective experiences described across different studies while acknowledging the variability in how CRCI is conceptualized and assessed.

Despite limited research, existing studies consistently highlight the detrimental impact of CRCI on quality of life (QoL) [8]. Qualitative research, particularly among survivors of breast cancer, has shown that patients often describe feeling less sharp, with impairments becoming evident when resuming everyday tasks [6]. As more individuals return to work following treatment, CRCI remains poorly addressed in occupational settings, with patients frequently reporting difficulties in planning, multitasking, and maintaining concentration. Nevertheless, remaining in employment has been associated with better QoL than withdrawal from the workforce, emphasizing the need for effective workplace management strategies [9]. Beyond employment, CRCI also disrupts interpersonal relationships, with language and communication deficits contributing to misunderstandings. Some individuals report that family members minimize their symptoms or undermine their authority, further exacerbating distress [10].

Currently, there are no effective treatments for CRCI, but interventions can help alleviate symptoms. Pharmacological interventions, such as antidementia drugs, have shown limited benefit; for example, a trial of donepezil in patients with breast cancer reported no significant improvements in memory or other domains [11,12]. Typically, management strategies such as physical exercise, mindfulness, and cognitive training are used [13]. Engaging in aerobic and strength training for 6 months has been shown to improve self-reported cognitive issues, fatigue, and QoL [14]. Management techniques such as note-taking, reminders, and dietary changes are widely used, but patients continue to express the need for alternative evidence-based treatments [15].

Despite numerous reports, CRCI remains underexplored across prevalent cancer types. Qualitative evidence regarding CRCI is insufficient, and there is no comprehensive understanding of the lived experience. This study differs from existing review papers because, to date, the patient experience of CRCI has not been formally explored. By examining qualitative and mixed methods evidence across multiple cancer types, it addresses important gaps in understanding how CRCI is perceived, experienced, and managed in daily life. The novelty of this study lies in its focus on patient experiences using qualitative data and its inclusion of a range of cancer populations beyond breast cancer, which have historically been underrepresented in CRCI research. These elements distinguish this study from the existing predominantly quantitative literature. The findings could identify specific cognitive domains, cancer types, or treatments disproportionately affected by CRCI, informing the development of tailored interventions for its management and maintenance.

### Objectives

The objective of this review is to synthesize qualitative evidence on the lived experience of CRCI across cancer types. This review aims to address the following questions: What is the experience of individuals diagnosed with cancer who report CRCI across different cancer types? How commonly is CRCI subjectively experienced and reported by patients across various cancer populations? Which cognitive domains do patients perceive as most affected by CRCI? How does the subjective experience of CRCI affect patients’ QoL and functional ability?

## Methods

### Design

A detailed protocol for this systematic review has been previously published, ensuring methodological transparency and adherence to established guidelines [16]. The systematic review and thematic synthesis of qualitative studies was conducted in compliance with the steps of Thomas and Harden [17]. This study is reported in line with the PRISMA (Preferred Reporting Items for Systematic Reviews and Meta-Analyses) guidelines [18] (checklist provided in Multimedia Appendix 1) and the Enhancing Transparency in Reporting the Synthesis of Qualitative Research (ENTREQ) statement [19].

### Eligibility Criteria

The PICo (population, phenomenon of interest, and context) framework was used to assess studies and to establish the inclusion and exclusion criteria [20].

### Population

The qualitative synthesis included papers involving patients aged 18 years and older who were either currently receiving or had completed cancer treatment and had experienced cognitive impairment. There were no restrictions regarding gender, tumor type, or comorbidities.

### Phenomenon of Interest

The phenomenon of interest in this study is the experience of CRCI. As no definitive term is used to describe CRCI across the literature, the following terms were included: CRCI, cognitive impairment, mild cognitive impairment, cognitive dysfunction, and cognitive decline. The definition of cognitive impairment aligns with the *APA Dictionary of Psychology*, which defines it as any impairment in perceptual, learning, memory, linguistic, or thinking abilities [21].

### Context

This qualitative synthesis did not impose any restrictions on the context of patient experiences with CRCI, encompassing all geographic locations. The contexts considered included community-based environments such as patients’ homes or residential care facilities, as well as primary care settings including, general practitioner offices, pharmacies, and hospitals.

### Inclusion and Exclusion Criteria

The inclusion criteria for this review were interviews, either structured or semistructured conversations with an interviewer; focus groups, defined as facilitated group discussions; only the qualitative components of mixed method surveys that included verbatim quotes; qualitative data exploring patient experience of CRCI; qualitative data extracted from mixed method studies; and studies published in English.

The exclusion criteria were gray literature; reviews; quantitative data, including questionnaires with scales or any data without verbatim quotes; the experience or perception of carers or practitioners; conditions other than cancer; and cases where there is no mention of cognitive impairment.

### Search Strategy

A literature search for qualitative and mixed methods studies was conducted using the online databases PubMed, APA PsycInfo, CINAHL, and Scopus. These databases were selected for their broad coverage of relevant research fields, aligning with the objectives of this review. A 10-year timeframe was applied to ensure the review captured the most relevant and up-to-date evidence while providing sufficient breadth to identify developments over the past decade. The review began in June 2023, covering the last 10 years (2013-2023), and the search was updated to include publications through July 2025.

A librarian was consulted to ensure comprehensive search term coverage and to avoid omissions. The search strategy was customized for each database to include a wide range of relevant studies. Specific search terms are detailed in Multimedia Appendix 2. The search terms were based on the PICo framework, focusing on the population of people with cancer and the phenomenon of CRCI and its experiences. No contextual conditions were specified; therefore, no such terms were included in the strategy.

### Data Extraction

The results from each database were uploaded to Rayyan [22], an AI-powered web platform by the Qatar Computing Research Institute, for systematic review management. The research team, consisting of 3 PhD students, 1 academic doctor, and 2 professors, brought a diverse range of expertise, ensuring a sound and methodical approach. Rayyan facilitated the deduplication process, collaborative screening of titles, abstracts, and full texts, and resolution of conflicts. Titles and abstracts were reviewed to select papers meeting the inclusion criteria. All screening was conducted in duplicate by 3 independent reviewers (MI, AM, and HM), with a third reviewer involved to resolve any disagreements. Subsequently, the selected papers underwent duplicate full-text screening by 4 independent reviewers (MI, HKR, RM, and MAP). To ensure consistency, 2 reviewers piloted the data extraction process by discussing and selecting 10% of the studies. This ensured that data extraction adhered to PICo guidelines, covering population, phenomenon of interest, and context. The finalized papers were uploaded to NVivo (version 1.3, build 535; QSR International), for comprehensive data synthesis [23]. The data extraction table recorded details such as author, publication year, geographic location, study design, data collection methods, cancer type, affected cognitive domains, management strategies, and potential themes. References were managed using EndNote (Clarivate).

### Data Synthesis

The results section was imported into NVivo for data synthesis, following the 3-step thematic synthesis methodology outlined by Thomas and Harden [17]. First, each included paper’s results section underwent line-by-line coding to identify key concepts, which were then translated across studies to understand patient experiences of CRCI. Second, similar codes were grouped into descriptive themes based on the verbatim data from the selected studies, with a review process ensuring accurate data interpretation. Finally, inductive reasoning was used to develop analytical themes from the descriptive themes, capturing the patient experience of CRCI and aligning with the objectives of this qualitative synthesis. An independent reviewer (MI) conducted the interpretation of these analytical themes.

### Quality Appraisal

The quality assessment of the included studies was carried out using the Critical Appraisal Skills Programme (CASP) checklist, as recommended by Cochrane for qualitative synthesis [24]. Two independent reviewers (MI and HKR) applied the CASP tool to evaluate the quality of 10% of the studies (n=5). The remaining studies were appraised by the main researcher (MI). Any disagreements between reviewers were resolved through discussion, and if significant disagreements persisted, an additional reviewer was consulted until consensus was reached. The included studies were positively appraised by the CASP tool, indicating sound methodology and supporting the credibility of the findings.

## Results

### Summary of Included Studies

**Figure 1:**
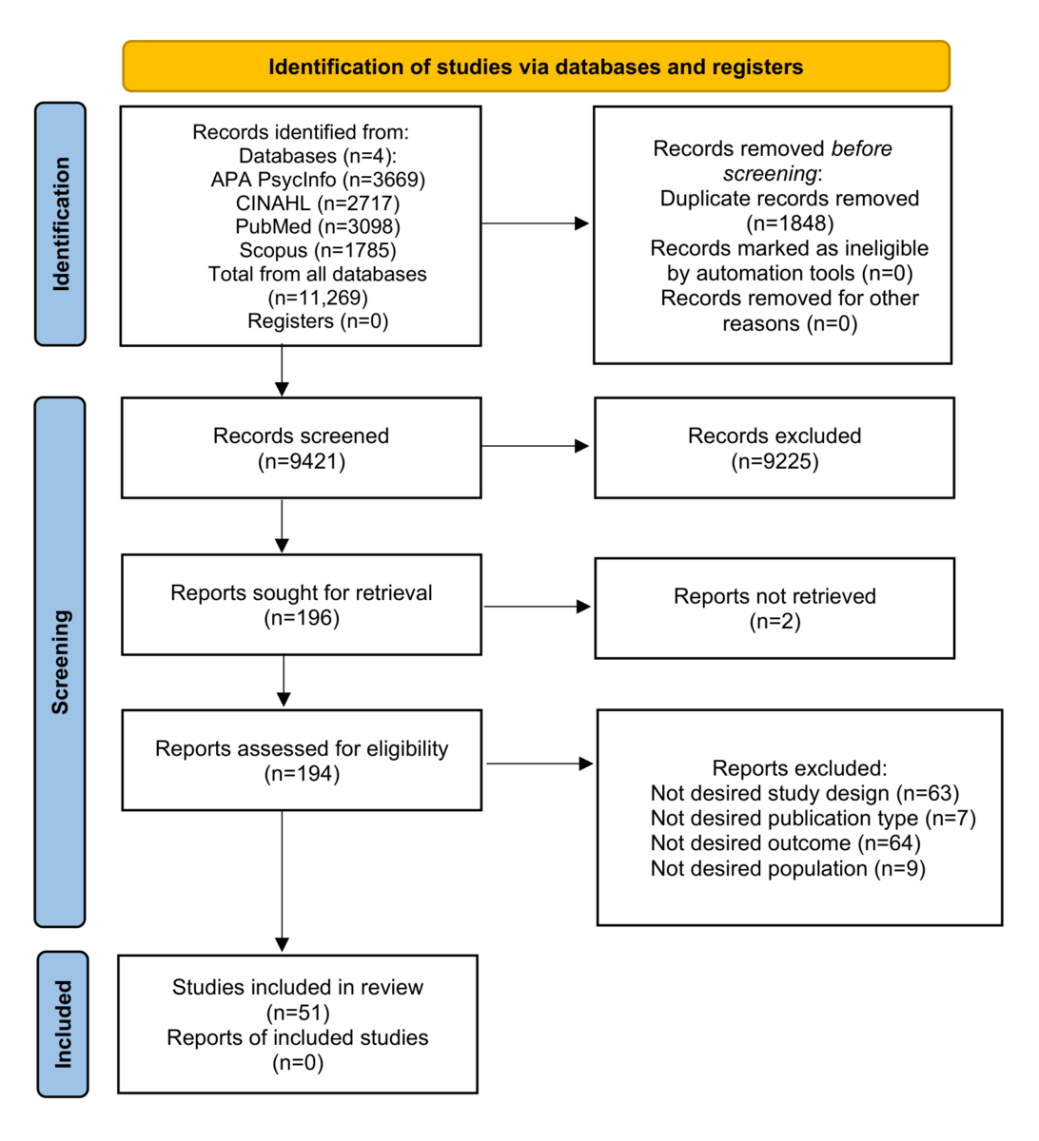
PRISMA (Preferred Reporting Items for Systematic Reviews and Meta-Analyses) flow diagram of search and study selection.

Figure 1 presents the results of the search and review strategy using the PRISMA flow diagram [18]. The search produced 11,269 articles, and following removal of duplicates (n=1848), a total of 9421 studies were included in the title and abstract review. Full-text review was performed on 194 studies, of which 51 were retained. Reasons for exclusion are provided in Figure 1.

### Description of Included Studies

Of the 51 papers included, the majority originated from the United States (n=20) [25-43], followed by Australia (n=8) [44-51], Canada (n=7) [52-58], Netherlands (n=4) [9,59-61], United Kingdom (n=3) [6,8,62], and China (n=2) [63,64]. Denmark [65], Hong Kong [66], Ireland [67], Turkey [68], Israel [69], South Korea [70], and Japan [71] each contributed 1 paper. This diverse geographical representation provides a broad perspective on CRCI across various cultural and health care contexts. Both qualitative (n=37) and mixed methods (n=14) papers were included. Further study information is provided in Table 1, which presents the frequency of cancer types identified across the included studies. Breast cancer was most frequently reported to be impacted by CRCI, followed by leukemia, colorectal cancer, and non-Hodgkin lymphoma.

**Table 1:** Prevalence of CRCI^a^ across cancer types

Cancer type	Papers reporting cancer type, n
Breast cancer	32
Leukemia	8
Colorectal cancer	8
Thyroid cancer	3
Non-Hodgkins Lymphoma	3
Lung cancer	3
Hodgin’s lymphoma	3
Testicular cancer	2
Prostate cancer	2
Melanoma	2
Head and neck cancer	2
Cervical cancer	2
Bladder cancer	2
Vulva cancer	1
Multiple myeloma	1
Liver cancer	1
Kidney cancer	1
Hematopoietic stem cell cancer	1
CNS^b^ cancer	1
Bowel cancer	1
Bone cancer	1

^a^CRCI: cancer-related cognitive impairment.

^b^CNS: central nervous system.

### Thematic Analysis

The analysis revealed 4 primary themes and 16 subthemes, which are presented in Table 2. Theme 1, cognitive challenges, involved issues with memory, concentration, executive functions, language, and processing speed, significantly impacting daily life. Theme 2, navigating employment, addressed the complexities of returning to work, disclosing the condition, workplace limitations, and the level of employer support. Theme 3, emotional, behavioral, and psychological impacts, included emotional responses, behavioral changes, and psychological effects. Theme 4, support systems, encompassed social support, health care professional (HCP) support, the timeliness of information, and attribution of symptoms.

**Table 2:** Themes and subthemes.

Theme and subtheme		Quotes
**Cognitive challenges**		
	Memory	“Remembering things is a little foggy, but if somebody brings up a conversation, I can remember the majority of it. But if somebody asks me something, sometimes, it’s there. Sometimes, it’s not, so I guess recent memory is not really there, but long term is pretty good.” [Participant 61]
	Concentration	“[The nurse] showed me how to use [the suction machine], but I forgot. I used to be okay with [technical] stuff, but since [treatment], it's all gone. I don't have the concentration…I can't get my mind to it.” [Participant 67]
	Executive functions	“The other day I asked my son, “Where is my telephone?”— and I was talking on it! I know I never did anything like that before!” [Participant 35]
	Language	“When I was reading books, I saw the words, but I could not make sense of the words, even when I read the same sentence several times.” [Participant 64]
	Processing speed	“[I have] this confusion: Should I give a piece of cake? Or should I put salt into the soup or sugar instead? These are the kind of things that come up. I have to concentrate a lot when I do things, because I know, else I will mess it up!” [Participant 59]
**Navigating employment**		
	Return to work	“I already had a complex job before, but since coming back in February I have to concentrate really hard to get my brain to think on multiple levels and do multiple tasks. Since February, it's been very hard. I don't feel like I am back to before” [Participant 46]
	Workplace limitations	“At work, I would not say I have changed anything, but I am always… I am always trying to jot things down because I might be doing something at work and I think of something I need to do…actually, this is when I make mistakes a lot of the time when something comes into my mind that I need to do and I do not make a note of it and then just forget it and then I do not do it because I have forgotten it.” [Participant 8]
	Condition disclosure	“made a conscious decision early on I was not going to reveal to anyone in my industry. And it’s tough cause you kind of like, have your colleagues, your family and friends, and then sometimes you get shades of grey. So the shades of grey was the toughest part. I wrestled and wrestled with that, you know like my friends from work.” [Participant 33]
	Employer support	“After I became ill, the employer provided staff to share my work tasks...let me arrange my working hours according to my own physical condition, going or not going to workplace, also I decided how long I worked.” [Participant 63]
**Emotional, behavioral, and psychological impacts**		
	Emotional	“it is all very frustrating to not be able to remember just simple things that I could before. I just feel stupid sometimes when I can’t remember anything” [Participant 38]
	Behavioral	“I’ve just been terrified that people will find out that, that I’m not cognitively where I used to be because that’s what I live on . . . I try to take notes, but I cannot take enough notes to capture everything that I used to be able to remember just in my head.” [Participant 25]
	Psychological	“I was the one who was able to remember everything. And now no one can count on me anymore. It influences my self-confidence. [It is] like a sorrow. It is another thing that the illness has destroyed.” [Participant 65].
**Support systems**		
	Social support	“He [husband] took care of the dog. He did the grocery shopping. He was the house manager, basically just poured love and had a really good attitude, just “we are going to get through this and it’s going to be fine.” [Participant 32]
	Health care professional support	“I mentioned it to [my doctor], but I knew it was just one of those things I would have to cope with, so I just did.” [Participant 26]
	Information timeliness	“As soon as I started getting fuzzy, I started googling chemo brain and all that kind of stuff … and at that time it was still kind of very wishy-washy about whether it existed or not. I don’t know if that’s … I don’t really google it anymore … because I know that I have it and I don’t care what anyone says.” [Participant 6]
	Attribution challenges	“I have no idea whether it’s associated with my age or with my treatment.” [Participant 51]

#### Theme 1: Cognitive Challenges

The influence of various cognitive impairments on daily functioning was a predominant subject across all reviewed papers. The subsequent subthemes demonstrate the repercussions of each impairment on different facets of everyday life. Overall, this theme illustrates the frustration experienced by people with cancer as they grapple with the efficient and accurate completion of tasks. Additionally, it shows how such struggles can result in diminished independence and heightened vulnerability to risks.

#### Memory: “Not as Sharp”

The most frequently reported cognitive decline was memory loss, which often induced feelings of aging and involved difficulty recalling everyday tasks that were previously familiar. Issues with short-term memory were particularly prevalent in these reports.

My memory in general just seems to not be as sharp as it was. People would mention a conversation that we had maybe a week ago, a month ago; sometimes I wouldn’t remember talking to that person at all.

Participant 38

People with cancer often reported struggling to recall recent conversations despite being prompted by others. This reflects a broader difficulty with retaining new information and maintaining social relationships. They also often frequently mentioned a noticeable decline in memory compared with their previous abilities. Memory impairments were often persistent and severe, sometimes likened to dementia [53]. The enduring presence of these symptoms raises concerns about the possibility of a more serious condition, highlighting the need to take CRCI seriously.

#### Concentration: “Off With the Fairies”

People with cancer frequently reported a decline in concentration, which adversely affected their capacity to maintain focus. This impairment significantly hindered their ability to engage in activities such as cooking and using essential medical equipment critical for their ongoing treatment.

Trying to stay focussed on something was impossible, just oﬀ with the fairies. I felt like I wasn’t concentrating as much. I’d want to move onto something else other than what I was doing.

Participant 44

In this case, they did not feel present and often became restless. The inability to remain focused on a single task was a common issue that hindered efficiency and the completion of daily activities. This can lead to feelings of anxiety and frustration and a reduced QoL. Stress also exacerbated cognitive symptoms.

#### Executive Functions: “Making Wrong Turns”

The inability to execute formerly routine tasks was repeatedly mentioned, highlighting impairments in executive functions critical for activities of daily living. Examples included challenges with driving, using a phone, and cooking, thus demonstrating the breadth of tasks affected by cognitive decline after cancer treatment. These deficits not only hinder practical functioning but also emphasize the negative impact of CRCI on autonomy and QoL.

I have trouble with directions now, and just orientation when I’m driving; getting lost very easily, making wrong turns.

Participant 37

Deficits in spatial orientation, planning, and problem solving are reflected here. These cognitive lapses, although not severe, disrupt daily activities and contribute to feelings of anxiety and uncertainty.

cooking … I sometimes wonder what things are used for. I took out soup stock and remembered that, oh yeah, I was thinking of making miso soup. It has been like this a lot lately.

Participant 71

Challenges in recalling intentions and understanding the use of ingredients were described, indicating impaired cognitive flexibility and working memory. These cognitive difficulties could potentially affect overall independence and self-confidence.

#### Language: “A Fog That Was Blocking the Word”

Language processing difficulties were also frequently reported by people with cancer. The inability to read and write as fluently as before was distressing and discouraging. Verbal fluency impairments were also reported, such as difficulty forming sentences. This highlighted the disruption of CRCI in the daily lives of people with cancer, particularly regarding reading and text comprehension. The inability to make sense of words was also described.

Word recall is extremely diﬃcult. So when [I] would try and put a sentence together, it took everything out of [me] to concentrate to come up with the words and quite often [I] would end up using a diﬀerent word because [I] couldn’t think of the word that [I] needed to use. Like a fog that was blocking the word.

Participant 44

Significant difficulty in word recall and the need for word substitution were reported. One participant likened the struggle to find words to a fog, in which the word seems within reach but remains obscured, making it difficult to grasp clearly.

#### Processing Speed: “It’s Like Driving Drunk”

The issue of processing capabilities emerged prominently as a concern. People with cancer experiencing cognitive impairment often encounter difficulties in effectively managing daily activities. Basic tasks have become mentally taxing and require heightened attention. These challenges encompass tasks ranging from those with relatively minor repercussions, such as using the wrong ingredients, to those carrying significant potential risks, such as challenges associated with driving, thus highlighting the dangers of living under such cognitive constraints.

I mean it’s one thing at a time really, and it takes me 10 times as long to do something as it did.

Participant 31

Slower processing speed threatens their independence, as even routine actions become time-consuming and mentally exhausting. This reflects a loss of efficiency and increased cognitive strain, affecting both productivity and QoL. It highlights the frustration and difficulties experienced by people with cancer as their cognitive abilities decline.

#### Theme 2: Navigating Employment

This theme examines the impact of CRCI on employment. It addresses considerations regarding the decision to return to work, disclosure of the condition, employer support, and limitations encountered in the workplace. The studies reviewed predominantly focused on females with breast cancer; therefore, the experiences discussed are primarily based on this population.

#### Return to Work: “I Couldn’t Go Back”

People with cancer frequently reported decisions to abstain from re-entering the workforce. This reflects a broader struggle with cognitive deficits affecting employability and career continuity.

I couldn’t remember things so I couldn’t go back to work.

Participant 39

This statement highlights the significant impact of CRCI on employment, emphasizing the role of memory loss as a critical barrier to returning to work. The inability to recall necessary information impedes job performance and may undermine confidence in professional capabilities. Lack of confidence in returning to work was also frequently mentioned.

I’m not ready to come back into the practice; I don’t have any conﬁdence whatsoever about going back into medicine right now. That’s very disheartening and very unnerving to me.

Participant 38

In this instance, a medical practitioner felt unable to resume professional duties due to CRCI and lack of confidence. This case illustrates the negative impact of CRCI on careers, particularly those requiring high cognitive function, and demonstrates significant financial ramifications. The inability to work can lead to substantial economic hardship, exacerbating the overall burden on people with cancer and contributing to a diminished QoL.

#### Workplace Limitations: “I’m Just Not Getting This”

The most frequently reported impacts of CRCI among people with cancer were the adaptations and struggles encountered in the workplace. Many reported that performing their occupational duties had become considerably more challenging compared to their precancer experience, specifically due to increased reliance on making notes and a decline in in efficiency and effectiveness. These cognitive difficulties often necessitated critical decisions, such as reducing professional responsibilities or downgrading positions. This shift in work capabilities highlights the influence of CRCI on professional lives and overall well-being.

Additionally, the struggle to learn new employment-related skills was reported, further highlighting the cognitive obstacles faced in professional environments.

And I had to learn a lot of information that I just was not familiar with. So, I didn’t feel like, sometimes I would sit at my desk and think I’m just not getting this, I just you know, this is just not sinking in and I’m reading this over and over.

Participant 29

The cognitive barriers presented by these struggles highlight the broader implications for professional performance and job satisfaction. These persistent challenges at work can lead to significant demotivation, as people with cancer find themselves unable to keep pace with the demands of their roles. Concern about potentially having to downgrade at work reflects a threat to professional identity and financial stability.

#### Condition Disclosure: “Not Up to Snuff”

The issue of disclosure emerged frequently, with diverse perspectives. While some people with cancer feared stigmatization and chose not to disclose their CRCI, others felt compelled to disclose to preempt potential questions regarding their performance.

Am I going to be the next candidate for the next promotion? So, even if I get the job, can I keep it? […] There are so many roadblocks to not disclosing, and once you disclose, you stigmatise yourself.

Participant 52

This quote highlights the impact of both disclosing and not disclosing CRCI. The participant felt that discussing their condition would lead to differential treatment, while nondisclosure might hinder chances for promotions and career progression. However, others felt that disclosure alleviates pressure.

There were some that I worked with more closely that I felt like I had to tell because I didn’t want them to think I was dropping the ball you know ‘what the heck is going on with her you know, she’s not up to snuff lately.’ So there were some folks that I did feel like I needed to tell some colleagues so that they would hopefully be understanding of why I wasn’t producing like I had been before.

Participant 33

The participant felt compelled to inform close colleagues about their CRCI to prevent being perceived as incompetent, hoping they would be understanding of reduced productivity. This situation raises concerns about employer and colleague perceptions. The fear of judgment, whether disclosing or not, was frequently mentioned, highlighting the difficult decision people with cancer face in managing their condition at work.

#### Employer Support: “Can You Handle It or Not”

Employer support emerged as a minor yet significant subtheme in the context of returning to work posttreatment. This support facilitated a smoother transition back into the workforce for some. However, this positive experience was not universal. Several participants reported that their employers exhibited a lack of concern for their well-being. The perception of people with cancer as outwardly healthy, despite ongoing cognitive impairments, often led to a lack of workplace accommodations that might otherwise be provided to someone with visible signs of illness.

Little by little, the workload assigned to others was reassigned. They said because you looked so good, people around me thought you could work just as before…

Participant 63

The issue of appearing healthy while experiencing ongoing health struggles is demonstrated here. Because they seem outwardly fine, people with cancer often face increased task assignments. Conversely, some felt significantly supported by their employers.

We often had meetings, almost weekly, in which we shortly, it doesn’t have to be long conversations, just like how are you, can you handle it or not, take care of yourself, you don’t have to.

Participant 59

In this case, regular meetings were held with the employer to check in and discuss any possible issues pertaining to CRCI. This support allowed the participant to return to work and remain employed, facilitating career progression and mitigating potential financial issues associated with leaving work. It also highlights the importance of proper occupational health checks and workplace adaptations to enable people with cancer to return to work with confidence.

#### Theme 3: Emotional, Behavioral, and Psychological Impacts

This theme discusses the emotional, behavioral, and psychological impacts experienced by people with cancer with CRCI. It explores their feelings when coming to terms with and coping with CRCI. The various behavioral changes implemented to manage symptoms and the long-term impact on self-perception are also examined.

#### Emotional: “I Do Not Enjoy Life Anymore”

People with cancer frequently reported emotional distress associated with the awareness of CRCI onset. Noticing the decline of their previously healthy brain over time is “very distressing” [46]. While the symptoms themselves are upsetting to manage, noticing their onset and worrying about their impact is equally troubling. This can lead to a loss of self and feelings of despair.

I’m becoming more and more withdrawn. As if I am through with life. I do not enjoy life anymore. I’m not happy with anything. My unhappiness is increasing and increasing.

Participant 68

Living with CRCI can lead to extremely negative feelings, withdrawal from previously enjoyed activities, and overall poor life satisfaction. This highlights the emotional impact of CRCI from onset to management, which can result in depression and reduced QoL.

#### Behavioral: “One Thing at a Time”

Numerous behavioral changes aimed at managing and coping with CRCI symptoms were reported by people with cancer. The most frequently mentioned adjustment was increased reliance on notetaking. People with cancer often take notes to remember minor details, whether related to work or daily household tasks, which they previously did not need to document. Additional behavioral modifications included slowing down and segmenting tasks. They often mentioned that they “try to focus on one thing at a time” [46] and no longer pressured themselves to complete all tasks as they did before. Instead, they paid close attention to their physical and mental cues and adjusted their activities accordingly.

Another notable change involved the use of brain-training applications featuring tasks such as sudoku. One participant specifically reported improvements in concentration using these apps.

I seem to think that I can concentrate for between 10 and 20 minutes longer than I did previously. I do not know why I think it is specifically 10 to 20 minutes longer, but… I do not know I've got this idea from somewhere maybe I have loosely timed.

Participant 8

Engaging in mentally stimulating activities daily was also found to be beneficial. Such activities not only helped alleviate symptoms but also improved the ability to perform routine tasks more effectively. By incorporating these activities into their daily routines, people with cancer experienced enhanced cognitive functioning and better overall management of CRCI symptoms.

#### Psychological: “I Can’t Rely on My Brain”

The psychological impacts of CRCI were also extensively reported. Although many described adopting a positive mindset as a coping mechanism, some reported experiencing negative self-perception and a significant loss of confidence.

The importance of remaining hopeful was frequently mentioned as a strategy for coping with CRCI and accepting the progression of their cognitive impairment. One participant with cancer expressed this sentiment, stating:

I say all these problems will one day go away; I always think positive.

Participant 68

Despite the prevalence of optimism, the adverse effects on self-perception cannot be overlooked. There were frequent reports of the detrimental impact of CRCI on confidence and reliability.

The idea that I can’t rely on my brain as much is actually super upsetting; emotionally, it goes to my sense of identity, my sense of self, it makes me worry about my future.

Participant 49

This highlights the psychological turmoil they face, emphasizing the deep emotional impact that cognitive impairments have on their sense of identity and prospects.

These accounts reflect the complex psychological challenges associated with CRCI, juxtaposing the struggle with negative self-perception against a strong will to remain hopeful. Efforts to maintain a positive outlook amid cognitive challenges highlight their resilience.

#### Theme 4: Support Systems

This theme explores the various sources of support in place for people experiencing CRCI. It discusses social support offered by family members or peer support groups. HCP support, information timeliness, and challenges related to symptom attribution are also addressed in this theme.

#### Social Support: “It’s Ok”

The presence of a robust support network was frequently identified as an essential coping mechanism for managing CRCI. Many reported receiving indispensable support from spouses, while others highlighted the benefits of engaging with fellow people with cancer.

[Now] I leave the daily organisation to my husband … Just tell me what I have to do and I’ll do it, but don’t ask me to decide!

Participant 50

This reliance on a partner for daily task management was deemed highly beneficial, as it alleviates the cognitive burden of decision-making while experiencing CRCI. The role of peer support groups was also emphasized as a significant source of comfort and understanding. People who attended support groups shared positive experiences.

When I met fellow survivors at BCF (Breast Cancer Foundation) … yeah … I thought, they also experienced what I have experienced. So it’s ok. It’s not too bad and we laughed about it.

Participant 6

Interacting with others undergoing similar struggles provides a unique sense of relief and relatability that cannot be easily replicated by those outside the cancer community. This shared understanding and mutual support enabled participants to manage their symptoms more effectively and improve their QoL.

#### HCP Support: “Doctors Don’t Always Have Time”

A significant concern among people with cancer was the lack of support from HCPs. The primary issue regarding HCP support was its noticeable absence. Participants reported often refraining from discussing cognitive impairments during medical appointments, anticipating that such discussions would be of no benefit.

I probably wouldn’t discuss it with a doctor, to be honest. I might mention it, but doctors don’t always have time for these… side issues, I guess. They’re more concerned about cutting it out or making you well, as opposed to how you cope with life.

Participant 49

These statements highlight the perception that cognitive symptoms are not regarded as a priority by health care providers, who are primarily focused on immediate medical treatment and physical recovery. Participants felt that cognitive symptoms were not taken seriously or prioritized, likely due to the severity of the cancer they had endured. This lack of validation and support for managing cognitive impairments left many feeling neglected and unsupported. The failure to address cognitive symptoms highlights a critical gap in the care of people with cancer.

#### Information Timeliness: “It Hit Me Like a Boom”

People with cancer were rarely informed about the existence of CRCI before undergoing treatment and were often left to independently learn about it once symptoms manifested. This initial lack of awareness frequently resulted in shock and panic; however, as participants gradually acknowledged these cognitive deficits, they expressed an increasing need for validation of their experiences.

I didn’t even know my body was going to go through that. It hit me like a boom.

Participant 26

These accounts describe the sudden and noticeable onset of symptoms, forcing people with cancer to seek information independently to confirm the existence of their condition and validate their experiences. The lack of timely information regarding CRCI negatively impacted them. Without previous knowledge, participants were unprepared for the cognitive challenges accompanying treatment, leading to unnecessary distress.

#### Attribution Challenges: “Is It the Cancer?”

A recurrent point raised by people with cancer was uncertainty regarding the source of their CRCI. Many approaching older age found it challenging to accurately determine the cause of their symptoms. Additionally, this ambiguity was compounded by a lack of validation from friends and family. Some mentioned being told by loved ones that their cognitive impairments were merely a part of aging, as others of similar age also experience such issues. This feedback often made participants feel as though they were overreacting to their symptoms, leading them to refrain from seeking further support.

Is it the cancer? Or is it ageing? Or is it a disease?

Participant 25

This uncertainty reflects a broader lack of understanding of CRCI itself and may be related to inadequate support and validation from HCPs. This highlights the importance of technology-driven interventions to bridge these gaps in care.

## Discussion

### Overview

This study is the first qualitative synthesis of CRCI across multiple cancer types, offering unique insights into its prevalence and impact. Beyond identifying breast, leukemia, and colorectal cancers as most affected, it highlights underexplored challenges such as employment-related disclosure dilemmas, inconsistent workplace support, and the critical lack of pretreatment education and health care recognition of CRCI. Importantly, it also uncovers the resilience and coping strategies patients use, underscoring the need for broader research and more structured support systems. Overall, this review highlights the profound consequences of CRCI on QoL and the lack of understanding and resources in place to manage its effects.

An objective of this study was to understand how commonly CRCI is subjectively experienced and reported by patients across various cancer populations. This study found that breast cancer was the most affected, followed by leukemia and colorectal cancer. Global Cancer Observatory 2022 reported that breast cancer is the second most diagnosed cancer worldwide, comprising 11.6% of global cancer diagnoses, following lung cancer at 12.4% [72]. This predominance of breast cancer warrants further consideration. Breast cancer is a highly researched cancer type, leading to significant advancements in treatment and management, and therefore resulting in higher prevalence and reporting. Patient characteristics, such as younger age at diagnosis and the psychosocial impact of treatment, may partly account for the higher reported prevalence [73]. There is currently no evidence to explain the cause of this prevalence, highlighting the need for broader research across different cancer types to fully understand the scope and impact of cognitive impairments in people with cancer.

Impaired memory, concentration, executive functioning, language, and processing speed emerged as the primary reported cognitive deficit (theme 1: cognitive challenges). These cognitive domains are consistent with previous findings [4] and may be clinically useful when screening for and diagnosing CRCI [38]. Participants frequently reported considerable frustration, particularly stemming from short-term memory and concentration impairments. The frustration was also due to difficulty completing tasks efficiently and accurately. This emotional response aligns with the findings from previous studies [74], which reported similar experiences among people with cancer facing cognitive challenges.

Impaired executive functions also affected participants’ ability to perform everyday activities such as cooking, driving, and using a phone. These impairments included lapses in spatial awareness, which significantly diminished QoL. This is consistent with previous findings reporting the negative impact of cognitive impairments on daily functioning [75]. Additionally, declines in processing speed required more focus for basic tasks and hindered quick decision-making, potentially creating dangerous situations and reducing personal independence. This aligns with existing studies that found processing speed is negatively impacted in CRCI [76,77].

Employment was a prevalent topic and highlighted the challenges people with cancer face in the workplace (theme 2: navigating employment). People with cancer are increasingly younger due to advancements in treatment, and many still have significant financial responsibilities [78]. However, cognitive impairments often hamper their ability to work. Therefore, employment is an important consideration when screening for and managing CRCI.

CRCI adversely affected job performance and led to a marked lack of confidence. Previous research also reported the workplace adaptations found in this study, such as reliance on notetaking, using reminders, downgrading positions, and reducing responsibilities [57]. The dilemma of disclosing CRCI to employers was another notable issue; some hesitated due to fear of judgment, while others disclosed to avoid being judged for poor performance. Supportive work environments enabled some participants to remain in the workforce and facilitated career progression, while others lacked support. A previous review indicates that managers often lack awareness regarding the most effective strategies for facilitating the reintegration of people with cancer into the workplace. This lack of knowledge may account for the observed deficiency in supportive measures [79]. Furthermore, proper occupational health checks and workplace accommodations would enable people with CRCI to return to work with confidence.

Theme 3: emotional, behavioral, and psychological impacts highlighted that CRCI symptom onset was highly distressing, often leading to a loss of self. Previous studies also report a loss of confidence due to CRCI, diminishing life satisfaction [74]. Changes such as increased notetaking, slowing down pace, and paying attention to physical and mental cues were implemented. Memory support techniques are effective in managing CRCI [80]. Brain training exercises were not frequently used by or offered to people with cancer; however, they were reported as useful when implemented, with one participant noticing improvement in cognition. This builds on a previous review, which found that computerized brain training is efficient for CRCI, but further studies are required to evaluate its effectiveness as a treatment [81].

Psychological responses to CRCI were varied; many participants maintained a positive and motivated mindset, while others struggled with negative self-perception. The findings align with a previous review reporting that psychological distress is consistent with CRCI [82]. Despite these challenges, the strong will to remain hopeful amid distress was a recurring theme, highlighting the resilience of people with cancer coping with CRCI. Cognitive behavioral therapy is an effective technique that promotes coping strategies. Internet-delivered cognitive behavioral therapy has been found to be beneficial for reducing distress in patients with breast cancer [83] and could be a potential intervention to improve emotional responses to CRCI. However, there are mixed findings regarding its efficacy for improving cognition, and this requires further exploration in this context [84].

Social support mechanisms primarily involved reliance on spousal assistance and participation in support groups, which provided valuable peer-support (theme 4: support systems). However, formal interactions with HCPs often revealed a significant gap in support. Many people with cancer reported that HCPs did not address CRCI symptoms, considering them secondary to the primary concern of cancer treatment. One study observed that discussions about CRCI are often contingent on the awareness and knowledge of HCPs or are initiated by patients themselves. The study suggests that this challenge can be addressed by integrating a standardized factsheet into routine cancer care, which would provide essential information and improve communication on CRCI [85].

Regarding information timeliness, people with cancer were often inadequately informed about CRCI symptoms before their onset, leading to significant panic and a lack of preparedness when symptoms emerged. This lack of early information necessitated independent efforts by people with cancer to seek validation and understanding of their symptoms, highlighting a critical communication gap that needs to be addressed as part of pretreatment education. Previous research found that patients with breast cancer were rarely informed about CRCI before treatment and that HCPs could be more involved in its management [86].

Additionally, attributing cognitive difficulties to CRCI was challenging, with some perceiving their symptoms as a normal part of aging. Currently, there is no clear relationship between cognition and cancer treatments, and other factors such as depression, lack of sleep, and drug interactions could contribute to cognitive impairment [84]. This reflects a broader lack of awareness and understanding of CRCI, emphasizing the need for increased awareness and support within personal networks.

### Limitations

Due to the breadth of the literature, defining the search criteria proved challenging, resulting in the potential exclusion of relevant studies. Additionally, the search and selection criteria may have led to the omission of relevant papers published in languages other than English. This study does not account for varying cancer treatment effects and their impacts on cognition. Furthermore, due to the interpretative nature of qualitative research methods, potential bias must be considered when evaluating the findings.

### Implications for Practice and Research

In future practice, HCPs should provide timely information regarding CRCI to people with cancer. This includes explaining the potential symptoms, possible impacts, and various management techniques to better equip them for symptom management. Moreover, there should be increased validation of symptoms and heightened awareness of CRCI among HCPs to ensure comprehensive care [86]. A factsheet should also be implemented in clinical settings.

This study indicates that while interventions are not commonly used by patients with cancer, those who engaged were willing to do so. Further research is required to investigate the types and consistency of interventions offered.

Future research should also explore additional management techniques and assess the feasibility of computerized brain training as a treatment for CRCI. CRCI remains an underexplored area of study, necessitating further investigation, particularly regarding its underlying causes.

### Conclusions

This qualitative systematic review highlights the profound negative impact of CRCI on the QoL of people with cancer. The evidence consistently shows that memory, concentration, executive functioning, language, and processing speed, are severely affected. These impairments have far-reaching consequences on employment, representing a substantial barrier to maintaining occupational roles and financial stability. It is important for HCPs to increase their awareness of CRCI and implement standardized screening processes to ensure timely identification and management. Furthermore, this study highlights a critical need for continued research into effective interventions, such as computerized brain training programs, which could potentially offer relief and improve cognitive functioning. Addressing these gaps in knowledge and practice will be essential for enhancing the QoL of people with cancer and supporting their reintegration into daily life and work environments.

## Appendix

Multimedia Appendix 1 PRISMA (2020) checklist.

Multimedia Appendix 2 Search strategy.

## Abbreviations

CASP: Critical Appraisal Skills Programme
CRCI: cancer-related cognitive impairment
ENTREQ: Enhancing Transparency in Reporting the Synthesis of Qualitative Research
HCP: health care professional
PICo: population, phenomenon of interest, and context
PRISMA: Preferred Reporting Items for Systematic Reviews and Meta-Analyses
QoL: quality of life

## Data Availability

The data used in this study are derived from previously published studies, which are publicly available and cited in this article.
